# Glomus Tumor of the Finger Diagnosed on Bone Scintigraphy

**DOI:** 10.1055/s-0045-1802955

**Published:** 2025-02-20

**Authors:** Alecio F. Lombardi, Felipe Martinez, Jeremiah R. Long

**Affiliations:** 1 Department of Radiology, Mayo Clinic Arizona, Scottsdale, Arizona, United States

**Keywords:** glomus tumor, finger, bone scintigraphy, MRI

## Abstract

A 60-year-old woman with chronic pain in the left long finger, occasionally involving the ring finger and intermittently extending to the hand, wrist, and distal forearm, was referred for radiographs and a triple-phase technetium-99m methylene diphosphonate bone scintigraphy to evaluate for possible complex regional pain syndrome. Initial radiographs were normal. A three-phase bone scan revealed focal radiotracer uptake at the distal aspect of the long finger during the blood pool phase only, with normal blood flow and delayed phases, suggesting a hypervascular soft tissue tumor. Follow-up magnetic resonance imaging showed a hypervascular nodule in the nail bed of the long finger. The lesion was surgically resected, and pathology results confirmed the diagnosis of a glomus tumor.

## Introduction

A glomus tumor is a benign neoplasm of the glomus body, a contractile neuromyoarterial structure consisting of an arteriovenous anastomosis surrounded by a capsule that participates in temperature regulation. Although glomus tumors can be found anywhere in the body, they more commonly arise in the subungual region of the fingers. They present with a classical clinical triad of intense pain, local tenderness, and cold sensitivity. The diagnosis is usually achieved via ultrasound or magnetic resonance imaging (MRI), which typically demonstrates a hypervascular or hyperenhancing solid nodule in the fingertip associated with the nail plate. We present an atypical case of a 60-year-old woman with a glomus tumor of the finger, incidentally diagnosed on bone scintigraphy performed for the evaluation of complex regional pain syndrome. The patient experienced worsening pain and local tenderness involving the left long and ring fingers, initially thought to be neuropathic or autonomic in origin. MRI of the cervical spine and electrodiagnostic testing of the upper extremity were negative. Radiographs of the hands and fingers did not reveal any abnormalities. A three-phase bone scan showed focal increased technetium-99m methylene diphosphonate (Tc-99m MDP) uptake at the tip of the long finger on the blood pool phase only, with normal appearance on the flow and delayed phases. Doppler ultrasound demonstrated a hypervascular solid nodule located in the subungual region of the long finger. MRI confirmed a small subungual enhancing nodule. The patient underwent surgical resection, and pathology confirmed the diagnosis of a glomus tumor.

## Case Report

A 60-year-old woman with a past medical history of breast cancer status post mastectomy and radiation therapy complained of worsening pain in the tip of the left long finger. Previously, the pain had been intermittent and limited to the tip of the long finger. However, it had recently become almost constant, significantly worse at night, and at times involving the ring and small fingers, extending to the mid-palm, wrist, and distal forearm. She had been evaluated a few years earlier and treated for carpal tunnel syndrome with a splint, which did not alleviate the symptoms.

On physical examination, there was a slight bluish hue along the ulnar aspect of the nail bed of the long finger. There were no swelling, nodules, or deformity of the digit or nail plate. The finger was extremely tender to the touch along the ulnar border of the nail fold and distal phalanx. No tenderness was noted in the palm, wrist, or forearm. Given the prior history of mastectomy and radiation therapy, the lack of nodules in the fingertips and the radiation of pain to the hand, wrist, and distal forearm, a neuropathic or autonomic etiology was initially suspected, such as cervical compressive radiculopathy, brachial plexopathy, or complex regional pain syndrome.

**Fig. 1:**
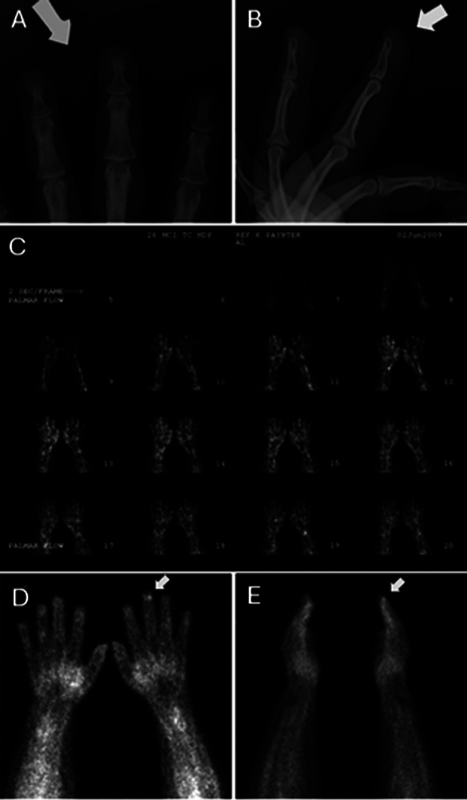
A 60-year-old woman with glomus tumor at the tip of the long finger. ( **A** ) Posteroanterior and ( **B** ) lateral radiographs of the long finger demonstrate no osteopenia, osseous lesions, cortical erosions, or soft tissue lesions. Bone scintigraphy following intravenous administration of Tc-99m MDP shows normal soft tissue perfusion in the dynamic flow phase ( **C** ) and focal radiotracer accumulation at the distal aspect of the left long finger in the blood pool phase ( *arrows* in **D** and **E** ). The delayed bone phase was normal (images not shown).

An MRI of the cervical spine was negative. Electrodiagnostic tests demonstrated no evidence of cervical radiculopathy, upper extremity neuropathy, or brachial plexopathy. Radiographs of the fingers did not reveal any signs of osteopenia, osseous lesions, cortical erosions, or soft tissue lesions. A three-phase bone scintigraphy was ordered to evaluate for complex regional pain syndrome. Following the intravenous injection of 740 MBq (20 mCi) of Tc-99m MDP, a dynamic blood flow phase, acquired during the first 60 seconds after the intravenous injection of the radiotracer, demonstrated normal perfusion in the hand and fingers. Similarly, delayed phase (3 hours) whole-body planar scintigraphic images were normal. However, the blood pool or soft tissue phase, obtained 5 minutes after radiotracer injection, demonstrated focal increased tracer uptake in the distal aspect of the long finger ( Fig. 1 ). These findings were suspicious for a highly vascularized soft tissue tumor, and an MRI was recommended for further evaluation.

**Fig. 2:**
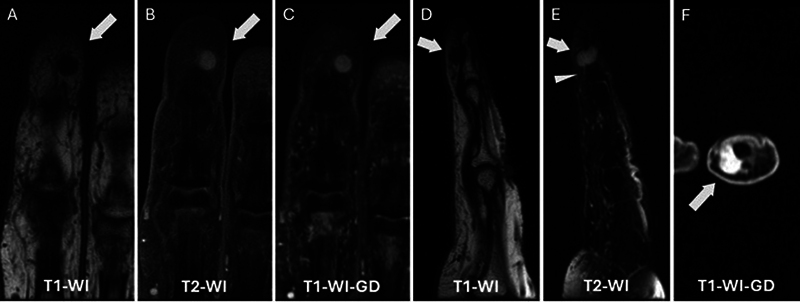
A 60-year-old woman with glomus tumor at the tip of the long finger. Magnetic resonance imaging of the long finger demonstrates a soft tissue nodule at the fingertip, extending from the subungual region to the volar aspect of the distal phalanx, with intermediate signal intensity on T1-weighted images ( **A, D** ), intermediate to high signal intensity on T2-weighted images ( **B, D** ), and diffuse homogeneous enhancement following intravenous gadolinium administration ( **C, F** ). Feeding vessels are visible on the sagittal T2-weighted images ( ***arrowhead* in E** ). GD, gadolinium; T1-WI, T1-weighted imaging, T2-WI, T2-weighted imaging.

The MRI revealed a well-circumscribed homogeneous, and diffusely enhancing lesion along the ulnar aspect of the fingertip, adjacent to the distal phalanx of the long finger, without evidence of cortical breakthrough ( Fig. 2 ). An ultrasound performed in the physical medicine office showed a solid nodule with increased vascularization on power and color Doppler in the subungual region of the distal phalanx of the long finger (images not available). All findings were consistent with a glomus tumor. A decision was made to proceed with surgical excision. During surgery, the tumor was located below the nail plate and nail matrix, adjacent to the distal phalanx. It was well encapsulated and bluish in color. Pathology, including histology and immunoperoxidase stains with diffuse immunoreactivity for α-smooth muscle actin (α-MSA) and muscle-specific actin (MSA), confirmed the diagnosis of a glomus tumor.

## Discussion

Glomus tumors are defined by the World Health Organization's classification of tumors of soft tissues and bone as mesenchymal neoplasms composed of cells resembling the modified smooth muscle cells of the normal glomus body. 1 The glomus body is a neuromyoarterial structure consisting of an afferent arteriole, an arteriovenous anastomosis, and an efferent venule, surrounded by a capsule of endothelial vascular spaces and glomus cells. Although glomus bodies can be found throughout the body, they are most commonly located in the dermis, particularly in the palms of the hands and soles of the feet. Their main role is to control temperature by contracting or dilating to preserve or dissipate heat, respectively.

Glomus tumors are well-circumscribed neoplastic proliferations of varying proportions of glomus, vascular, and smooth muscle cells. Depending on the dominant histological component, glomus tumors can be classified into the most common solid form (predominantly glomus cells), glomangiomas (predominantly vascular cells), or glomangiomyomas (predominantly vascular and smooth muscle cells). Occasionally, glomus tumors may show unusual, atypical, or malignant features based on histologic characteristics such as nuclear atypia, infiltrative growth, vascular involvement, necrosis, or mitotic activity. 2 Immunohistochemical analysis typically shows positivity for α-SMA and MSA, while being negative for CD31, cytokeratins, and S100. 3

Glomus tumors may be evident on direct visual inspection as a blue-hued nodule causing deformity in the nail bed or on the volar aspect of the fingertip. The classic clinical triad consists of severe pain, local tenderness, and cold sensitivity. The differential diagnosis is broad and includes lipoma, cyst, angioma, hemangioma, neuroma, melanoma, and gout. 3 Atypical presentations such as pain radiating to the hand or adjacent fingers, weakness, or prior history of surgery, radiation therapy, or trauma may further complicate the differential diagnosis, which also includes compressive radiculopathy, brachial or lumbar plexopathy, mononeuropathy, or complex regional pain syndrome.

In the current case report, the patient had a prior history of mastectomy and radiation therapy. The pain in the long finger was intermittent, at times involving the middle and little fingers, radiating to the palm, wrist, and distal forearm. Although there was a bluish discoloration at the fingertip, there were no obvious nodules in the nail bed or volar aspect of the fingers. The findings were initially thought to be secondary to a neuropathic or autonomic etiology, such as radicular neuropathy, brachial plexopathy, or complex regional pain syndrome, prompting initial investigations with MRI of the cervical spine, electrodiagnostic tests, and a three-phase bone scintigraphy. Given that glomus tumors are rare, high clinical suspicion by both the clinician and the radiologist is important to narrow the differential diagnosis.

On radiographs, cortical erosion may be seen if there is intraosseous extension. Ultrasound typically demonstrates a well-circumscribed, solid hypoechoic nodule with increased flow on power and color Doppler. MRI is highly sensitive in identifying glomus tumors, showing a solid nodule with intermediate signal intensity on T1-weighted sequences, intermediate to high signal intensity on T2-weighted sequences, and diffuse enhancement following gadolinium administration. 4 Small tortuous feeding vessels adjacent to the tumor can be identified both on ultrasound and MRI.

Tc-99m MDP three-phase bone scintigraphy demonstrates focal tracer accumulation in the soft tissues on the blood pool or soft tissue phase only, while the early dynamic blood flow and delayed bone phases remain negative, reflecting the highly vascular nature of the tumor. 5 This contrasts with the typical triple-phase positivity found in complex regional pain syndrome, where there is diffuse periarticular tracer accumulation in all three phases. Rheumatoid arthritis or gout would also show more periarticular and widespread tracer accumulation in all three phases, rather than focal uptake at the tip of the finger. Additionally, 18F-fluorodeoxyglucose positron emission tomography (18F-FDG PET/CT) is highly sensitive for detecting glomus tumors located in the deep soft tissues or internal organs. Some reports show increased FDG avidity in glomus tumors located in the musculature, trachea, or lungs. 4 6 7 8
